# Functional Traits Differ between Cereal Crop Progenitors and Other Wild Grasses Gathered in the Neolithic Fertile Crescent

**DOI:** 10.1371/journal.pone.0087586

**Published:** 2014-01-28

**Authors:** Jennifer Cunniff, Sarah Wilkinson, Michael Charles, Glynis Jones, Mark Rees, Colin P. Osborne

**Affiliations:** 1 Department of Animal and Plant Sciences, University of Sheffield, Sheffield, United Kingdom; 2 School of Archaeology, University of Oxford, Oxford, United Kingdom; 3 Department of Archaeology, University of Sheffield, Sheffield, United Kingdom; University of Toronto Mississauga, Canada

## Abstract

The reasons why some plant species were selected as crops and others were abandoned during the Neolithic emergence of agriculture are poorly understood. We tested the hypothesis that the traits of Fertile Crescent crop progenitors were advantageous in the fertile, disturbed habitats surrounding early settlements and in cultivated fields. We screened functional traits related to competition and disturbance in a group of grass species that were increasingly exploited by early plant gatherers, and that were later domesticated (crop progenitors); and in a set of grass species for which there is archaeological evidence of gathering, but which were never domesticated (wild species). We hypothesised that crop progenitors would have greater seed mass, growth rate, height and yield than wild species, as these traits are indicative of greater competitive ability, and that crop progenitors would be more resilient to defoliation. Our results show that crop progenitors have larger seed mass than wild species, germinate faster and have greater seedling size. Increased seed size is weakly but positively correlated with a higher growth rate, which is primarily driven by greater biomass assimilation per unit leaf area. Crop progenitors also tend to have a taller stature, greater grain yield and higher resilience to defoliation. Collectively, the data are consistent with the hypothesis that adaptations to competition and disturbance gave crop progenitors a selective advantage in the areas surrounding early human settlements and in cultivated environments, leading to their adoption as crops through processes of unconscious selection.

## Introduction

Grain assemblages from early settlements in the Fertile Crescent of Southwest Asia show that hunter-gatherers collected a large, diverse range of species before the origin of agriculture [1]–[8]. However, later assemblages in the region suggest a progressive specialization in diet, characterized by a decline in the occurrence of some wild plant species, and an increase of other species as they were brought into cultivation [5], [8], [9]. Domesticated crops emerged in the Fertile Crescent of Southwest Asia between ∼11,000 and 8,000 BP [10]–[12], and are characterized by the evolution of a ‘domestication syndrome’, including the loss of natural dispersal mechanisms and an increase in seed mass [13], [14]. However, it is not clear what drove the selection of some species as crops and what caused the abandonment of others.

The selection of crop species could have been an intentional selection process by early farmers in response to demographic [15] or social [16] pressures, or it could be the result of unconscious selection [17]–[20]. Unconscious selection arises from the interactions between humans and their food plants, and has the potential to determine the species that were domesticated and to drive the domestication process. Some authors have suggested a prolonged period of pre-domestication cultivation, during which certain species were cultivated but had not yet acquired the morphological changes associated with domestication [8], [21]–[23]. We propose an ecological model, whereby selection could have occurred in two distinct settings prior to the establishment of full agricultural systems, each introducing plant species to novel anthropogenic environments. Initially, the gathering of wild plants for subsistence and the accidental spillage of seeds may have concentrated food plants in the area surrounding settlements. Selection then acted on this local species pool via disturbance, competition and high soil fertility. Cultivation subsequently modified the selection regime through the deliberate sowing of seeds in tilled soil and, at some point, by other management practices such as weeding, manuring and irrigation. This paper explores the potential role of these ecological selection processes in the domestication of crops. We set up an experiment to compare the crop progenitors and co-collected wild species to investigate whether there are functional traits of these crop progenitors that would have favoured their selection in these early agricultural environments.

High relative growth rate (RGR), defined as the rate of dry matter production per unit of dry matter under optimal conditions [24], [25], is generally considered a crucial adaptation to fertile, disturbed niches. RGR represents one of the fundamental axes of ecological variation, corresponding with rapid rates of resource acquisition, and trading off against allocation to storage, defence and survival [25]–[27]. High RGR has previously been shown to correlate negatively with seed mass, suggesting that small-seeded species have growth strategies (physiological, morphological or structural adaptations) to facilitate rapid growth [28], [29]. However, because RGR typically declines as plants grow, comparisons of species with different seed sizes will inevitably confound size effects with true differences in growth strategy [30]–[32]. To separate the effects of size and growth strategy, Metcalf *et al.* [33] suggested making comparisons at a common size and, when this was done, a positive relationship between RGR and seed mass was found in short-lived species [34] raising the possibility that fast growth may be an important component of the domestication syndrome in large-seeded crops. Large seed size also correlates with a range of other traits that would be an advantage in early anthropogenic environments, including quicker and earlier germination [35], enhanced competitive ability [36] and survival following burial [37].

This paper tests the overarching idea that Fertile Crescent crop progenitors might be better adapted to disturbed, fertile environments than other wild grass species exploited by pre-agricultural societies by testing five hypotheses: (i) crop progenitors have larger seeds and seedlings, and faster germination than the other wild species; (ii) the RGR under fertile soil conditions is greater in crop progenitors than the other wild species, when compared at a common size; (iii) size and seed yield at maturity are greater in crop progenitors than the other wild species; (iv) resilience (survival and fecundity) of crop progenitors when defoliated is greater than that of wild species; and (v) the differences between wild species and crop progenitors outlined in (ii) to (v) can be explained by correlations of each trait with seed mass.

## Materials and Methods

Three experiments were designed to test the hypotheses. Experiment 1 investigated whether seed mass had an effect on the timing and rate of germination. Experiment 2 investigated the relationship between seed mass and growth rate using a functional approach with repeated harvests to calculate RGR using conventional and size standardised methods. The RGR data have been presented previously as part of a meta-analysis investigating the association of seed size, plant size and growth rate [34]. Here, the data are further analysed to determine how variation in plant allometry and physiological processes contribute to interspecific variation in RGR. The third experiment tested how crop progenitors and wild species respond to simulated disturbance via complete removal of above ground biomass, comparing survival and yield.

### Species selection

Nine species were selected for the experiments. All were grown from seed obtained from germplasm holdings, and different seed accessions were used for the three experiments (Table S1). To ensure that these accessions were reasonable representatives of the original progenitors, they were selected based on their region of origin, being as close to the centres of domestication as possible. While intraspecific variation and maternal effects are to be expected, previous experiments show that they are typically less important than interspecific variation. Species identity was ensured by the use of taxonomic and molecular markers to look for contamination in the germplasm holding centres [38]. The outer glumes were removed from all seeds before weighing. Since this study focuses on cereal cultivation, only grasses were chosen to ensure that comparisons between crop progenitors and wild species were not confounded by growth habit or phylogeny.

Three wild grasses, which became the major domesticates and staple foods in Southwest Asia were selected to represent the crop progenitors: *Hordeum spontaneum* Koch, *Triticum boeoticum* Boiss., and *Triticum dicoccoides* Koern. Six wild grass species that were never domesticated were also investigated: *Aegilops crassa* Boiss., *Aegilops speltoides* Coss., *Aegilops tauschii* Coss., *Eremopyrum bonaepartis* (Spreng.) Nevski, *Eremopyrum distans* (K.Koch) Nevski and *Taeniatherum caput-medusae* (L.) Nevski. These particular species were chosen because they are present in significant numbers in archaeobotanical assemblages, from numerous sedentary sites across Southwest Asia ranging in age from 23,000- 9,700 BP [1], [2], [4], [5], [7], [8], [10], [39], [40]. From the range of preserved plant remains, wild annual grasses were abundant at the majority of these early settlements and were chosen as a focus for the study.

For example, from the 90,000 plant remains collected at Ohalo II, an Upper Palaeolithic site in Israel, 19,000 were grass grains [4], [5]. The abundance of wild grasses at this location suggests they were a staple food source. Furthermore, there is evidence for the processing of plant material at this site, with a large concentration of remains around a grinding stone, and the presence of starch grains indicating the pounding and grinding of grass seeds [41]. Four sites in northern Syria show evidence of the collection of a wide range of grasses, with *Aegilops* spp., *T. caput-medusae* and *Eremopyrum* spp. being identified at all locations and *H. spontaneum* and *T. boeoticum* highly abundant in the majority of samples collected [8]. At Neolithic Çatalhöyük, east Turkey, two out of eight archaeobotanical samples from storage structures in burnt houses were dominated by *T. caput-medusae* accompanied by a lesser, but still substantial, amount of *Eremopyrum* type grass [7]. The purity of the samples, including one consisting solely of *T. caput-medusae* and *Eremopyrum* spp., suggests that seeds were being gathered, processed and stored.

### Experiment 1 – Seed mass and germination rate

#### (a) Plant material and growth conditions

Germination took place under propagator lids and a thin layer of wet compost, within a controlled environment chamber (BDR 16, Conviron, Winnipeg, Manitoba, Canada) under a 20/10°C day/night with an 8 hour photoperiod, PPFD of 300 µmol photons m^−2^ s^−1^, and humidity of 70%/50% day/night. Seeds were uncovered after two days to observe radicle length and then observed daily for germination. Seedlings were harvested when the ligule of the first true leaf emerged. Roots were washed free of compost, and seedlings were oven dried to a constant weight at 80°C before weighing.

#### (b) Statistical analysis

Regressions lines were fitted to plots of germination *vs* seed size, and seedling mass *vs* seed size. R^2^ values were calculated to estimate the goodness of fit and the significance of the relationship was tested using the *lm()* function in R (version 2.6.14, The R Foundation for Statistical Computing).

### Experiment 2 – Seed mass and RGR

#### (a) Plant material and growth conditions

Seeds were germinated on moist, washed sand (Chelford 52; WBB Minerals, Sandbach, Cheshire) under propagator lids in a controlled environment room (BDW 40, Conviron). Conditions were 20/10°C (day/night) with an 8 hour photoperiod and PPFD of 300 µmol photons m^−2^ s^−1^.

The general approach for growth analysis was based on studies by Grime and Hunt [25], Poorter and Remkes [42], Poorter [43], Hunt and Cornelissen [44] and Hendry & Grime [45]. Three days after germination, 24 seedlings of a uniform size were selected for each species and planted into 1 litre pots containing washed sand. These were top-watered every two days with full-strength Long Ashton nutrient solution ([46] Tables 40, 41), and bottom-watered with distilled water on alternate days. The seedlings were returned to the controlled environment room at: 20/10°C (day/night) with a 16 hour photoperiod, maximum PPFD of 756 µmol photons m^−2^s^−1^ and RH of 70%. Although these light levels are lower than the peak value in full sunlight, the levels are comparable to or higher than the irradiance levels used in most previous growth experiments measuring RGR and its growth components (see Shipley *et al.* [47]). For reference, the daily quantum input (DQI) in this experiment was 43.6 mol m^−2^ day^−1^.

#### (b) Harvesting schedule and measurements

The experiment was started 7 days after germination, when all plants had reached a fresh weight of approximately 100 mg (day 7). Harvests were carried out over a 3-week period on days 7, 10, 14, 17, 21 and 27. Each day, 4 plants of each species were harvested and divided into leaf blades, roots and leaf sheaths. Each plant was cleaned of the growth medium, and dried to a constant weight at 80°C for 2 days. The total leaf area of each plant was determined by scanning the freshly harvested leaf blades and using image analysis software (ImageJ 1.36b., Wayne Rasband, National Institute of Health, Bethesda, Maryland, USA).

#### (c) Statistical analysis

Regression lines were fitted to plots of seedling mass *vs* seed size, final dry mass *vs* seed size and final leaf area *vs* seed size. R^2^ values were calculated to estimate the goodness of fit and the significance of the relationship was tested using the *lm()* function in R (version 2.6.14, The R Foundation for Statistical Computing).

#### (d) Estimating RGR at a common size

A species-specific functional growth analysis was performed by fitting growth functions to plots of ln-total plant mass against time (Fig. S1), which were then used to estimate RGR at a common reference size for each species. For full details of the fitting and RGR estimation see references: [30], [32], [34], [48]. Fitting these curves and estimating RGR at a common size (sRGR) rather than calculating RGR via classical methods [44] accounts for size-dependent variation in growth rate bought about by changes in physiology, morphology and allocation. The 30^th^ percentile (0.067 g) of the distribution of plant mass was used as the reference size because it encompasses all the species, resource limitation should still be minimal, and growth approaching its maximum rate. Plant mass in the data set ranged from 0.005 g to 5.816 g.

RGR is determined by the net assimilation rate (NAR), which is the absolute growth rate per unit leaf area, and the leaf area per unit of plant mass, termed the leaf area ratio (LAR). The LAR is further factorized into: (1) leaf mass ratio (LMR), the proportion of biomass invested in leaves, and (2) specific leaf area (SLA), the leaf area per unit leaf mass. To better understand why RGR varies among species, the components of sRGR were calculated at the reference size (sNAR, sLMR and sSLA) based on predictions from linear regressions of ln-leaf area and ln-leaf mass against ln-total plant mass. In all cases the species × total plant mass interaction was significant (P<.05), so that all regression models had species-specific intercepts and slopes [30], [48].

### Experiment 3 – Components of yield and response to defoliation

#### (a) Plant material and growth conditions

Seeds were germinated in 24-cell plug-trays containing a 1∶1 sand: compost mix. Trays were placed in a controlled environment chamber (BDR 16, Conviron) under a 20/10°C day/night with an 8 hour photoperiod and a PPFD of 300 µmol photons m^−2^ s^−1^. Once the seeds had germinated and the seedlings had reached the second leaf stage, an 8-week vernalization treatment was imposed to enable flowering. Temperatures were set at 4°C (day and night) with PPFD and photoperiod as during germination. At the end of vernalization in late April, plants were re-potted into 4 litre pots containing the same growth medium. Plants were divided between three rooms in a glasshouse and organised in a randomised block design (Tapton Experimental Gardens, Broomhill, Sheffield). They were grown until maturity in mid-July. Artificial lighting was supplied by sodium lamps at 16 h d^−1^ throughout the experiment to supplement and extend natural daylight. The PPFD, temperature and humidity were logged at 5 minute intervals (DL2e data logger, Delta-T Devices Ltd., Cambridge, UK) to give mean maximum daily PPFD values of 420±31.1 µmol photons m^−2^ s^−1^, mean maximum daily temperatures of 24±0.6°C and mean minimum temperatures of 16±0.2°C. Mean maximum and minimum humidity values over a diurnal cycle were 62±0.8% and 43±1.6%, respectively. Over the full growing season, maximum daily temperatures ranged from 16°C to 35°C, maximum daily PPFD varied from 79 µmol photons m^−2^ s^−1^ to 830 µmol photons m^−2^ s^−1^, and maximum daily RH from 52% to 73%. Plants were watered every other day at the beginning of the experiment; this was reduced after flowering as the water demands of the plant decreased.

#### (b) Defoliation treatment and data collection

A defoliation treatment was applied to all nine species during the vegetative stage, two weeks after plants were transferred to the glasshouse by completely removing all plant material at 2 cm above the soil surface. There were eight replicates in the defoliated and unperturbed control treatments, giving a total of 16 individuals (8 replicates ×2 treatments) per species.

Measurements were made of six characters: survival (whether the plants flowered and set seed), time of flowering (days from end of vernalization period to extrusion of inflorescence on the leading tillers), plant height (measured from soil surface to the collar of the leading spike), the number of tillers (flowering and not flowering), number of seeds and potential yield. Survival and flowering were recorded whenever plants were watered. Plants were harvested after grain filling, but before spikes had started to shatter. Archaeological records show that prior to crop domestication, plants were harvested when grains were immature and still attached to the seed head, since the rachis of wild grasses shatters when mature, thereby dispersing the seeds [13]. Plant height and number of tillers (flowering and not-flowering) were recorded. The number of seeds on each plant was estimated by finding a relationship between the length of a spike and the number of seeds on a spike for each species. Grain yield of each species was estimated by multiplying seed number by the average values of seed weight measured in the RGR analysis.

#### (c) Statistical analysis

Data were analysed using the statistical computing package R (version 2.6.14, The R Foundation for Statistical Computing). All characters were tested using a generalized linear model (*glm*) with the appropriate error distribution and link function. In all cases the putative minimal model was determined by using the *dropterm()* function, and the best fit was determined by comparing dispersion parameters and AIC values. Comparisons were then carried out using a contrasts matrix [R-routine: *contrasts(Species)*] to determine whether there were differences in response to the disturbance treatment between crop progenitors and wild species.

## Results

### Seed mass and germination

**Figure 1:**
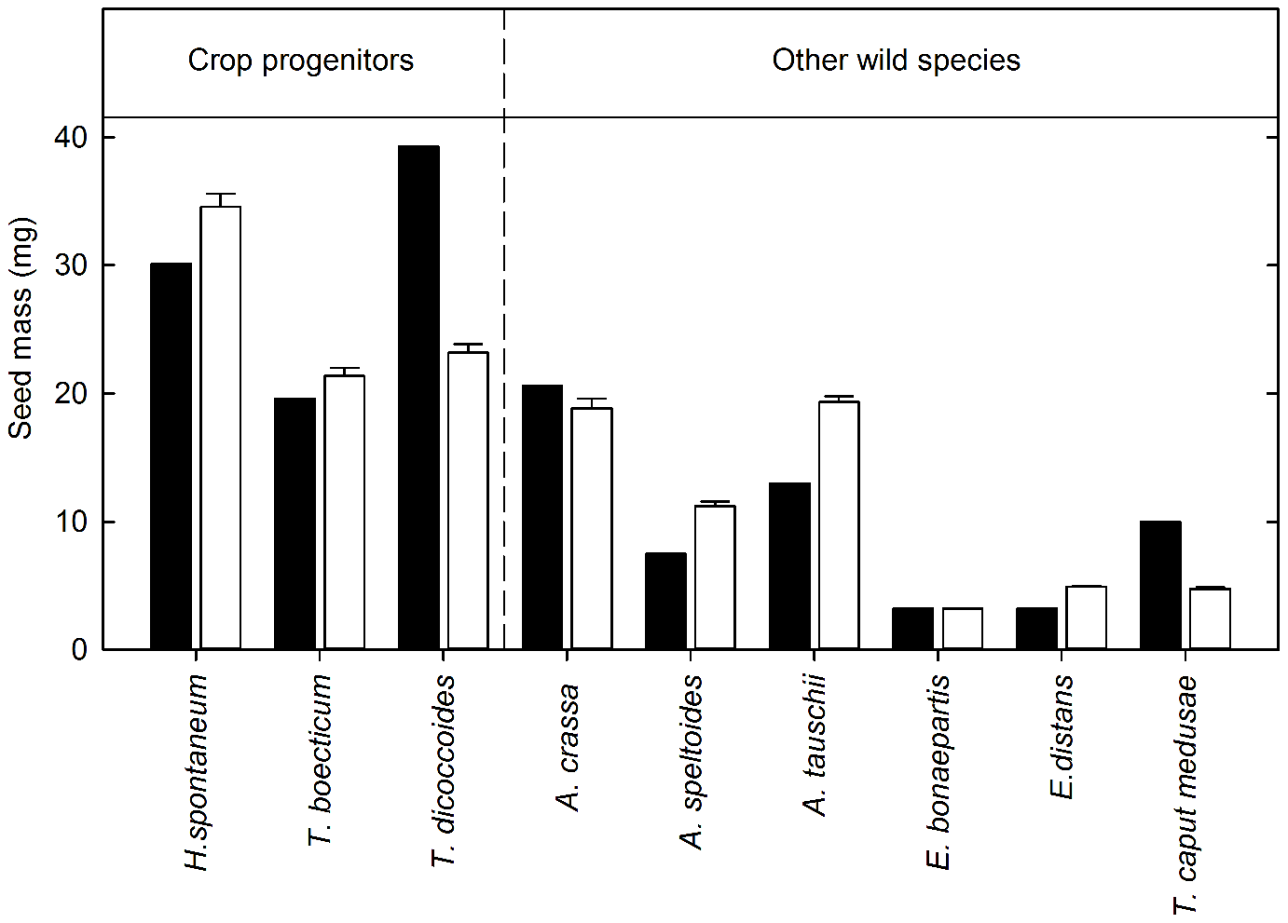
Initial seed mass in the three crop progenitors and six wild species. The black bars show the mean seed mass of accessions used in experiment 1 and the white bars show those used in experiment 2 (+SE). Standard errors are not shown for experiment 1 because seeds were not weighed individually.

Seed mass at the start of the experiment showed significant variation among species (F_8,63_ = 95.8, *P*<.001), ranging from 3.18±0.07 mg (mean±s.e.) in *E. bonaepartis* to 28.9±1 mg in *H. spontaneum* (Fig. 1). Seed mass was significantly larger in the crop progenitors than in the other wild species (t_1_ = 16.12, *P*<.001, Fig. 1). However, seeds of the crop progenitor *T. boeoticum* weighed 21±0.6 mg, only 10% larger than those of the two largest-seeded wild species, *A. crassa* and *A. tauschii*, which weighed 19±0.8 mg and 19±0.4 mg respectively. Seed mass differed between accessions (Fig. 1), but this variation did not obscure the strong overall difference between crop progenitors and wild species.

**Figure 2:**
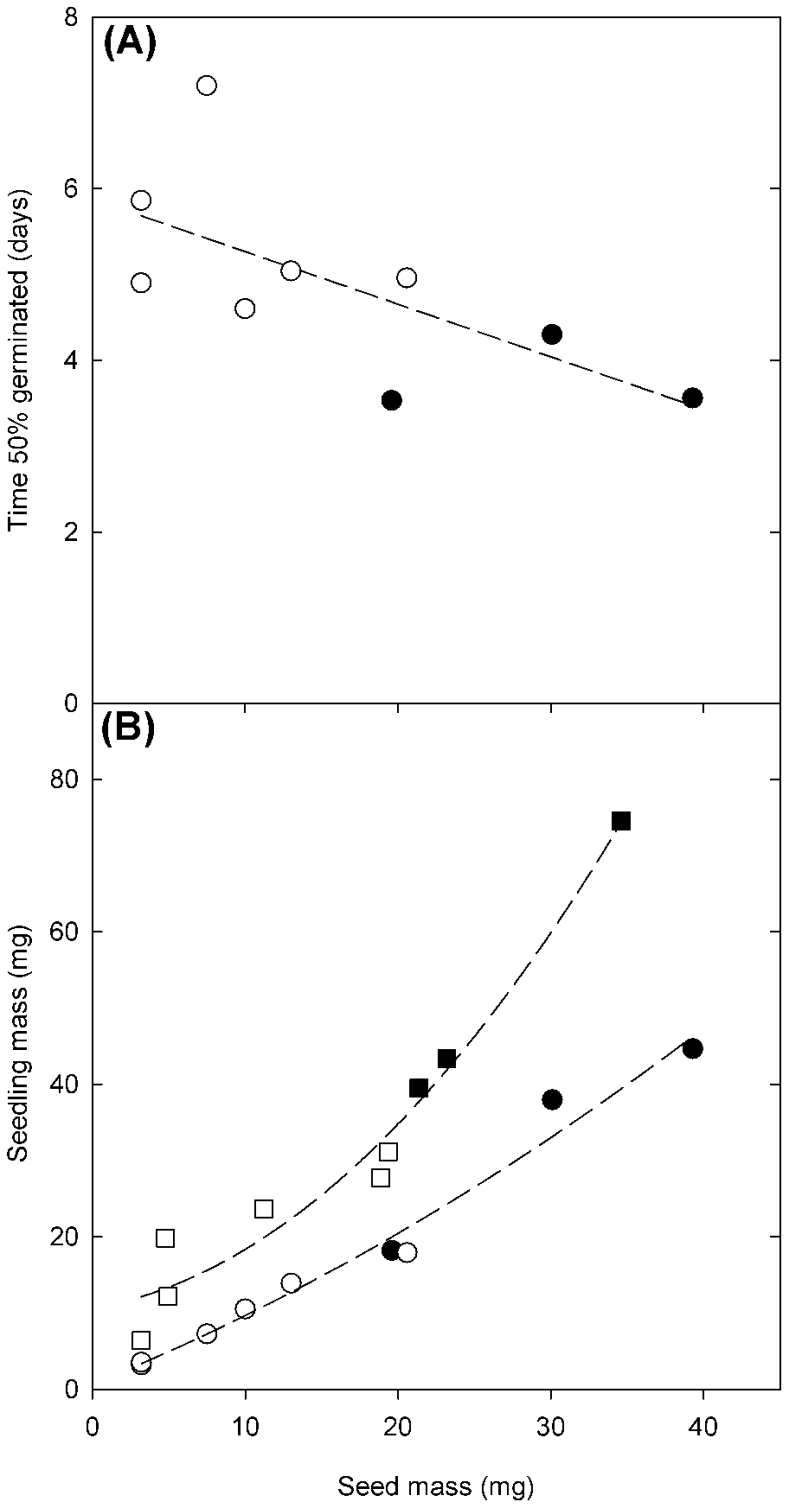
Relationship between seed germination, seedling mass and seed mass. Regression slopes for the relationship between (a) time to 50% of seeds germinated and seed mass (F_1,7_  = 5.55, *P*<.05, R^2^ = 0.44); and (b) seedling mass and seed mass, [experiment 1: circles (F_1,7_ = 120.156, *P*<.001, R^2^ = 0.9756), experiment 2: squares (F_2,6_ = 75.78, *P*<.001, R^2^ 0.9619)] for the three crop progenitors (closed symbols) and six wild species (open symbols).

Seed mass was negatively correlated with the time to germination, with larger seeded species germinating at a faster rate (F_1,7_ = 5.6, *P*<.05, Fig. 2A). Germination was approximately two days slower in the smallest seeded species (*E. bonaepartis*) compared to the largest seeded species (*H. spontaneum*). The larger seed mass in the crop progenitors meant that this group had a faster rate of germination when compared to the other wild species.

Larger seed mass was positively correlated with seedling dry mass in experiment 1 (F_1,7_ = 120.2, *P*<.001, Fig. 2B) and dry mass at seven days after germination in experiment 2 (F_2,6_ = 75.8, *P*<.001, Fig. 2B). This seed mass effect also carried through to the end of experiment 2, as shown by the positive correlations between dry mass and leaf area at 27 days (Fig. S2).

### Seed mass and RGR

**Figure 3:**
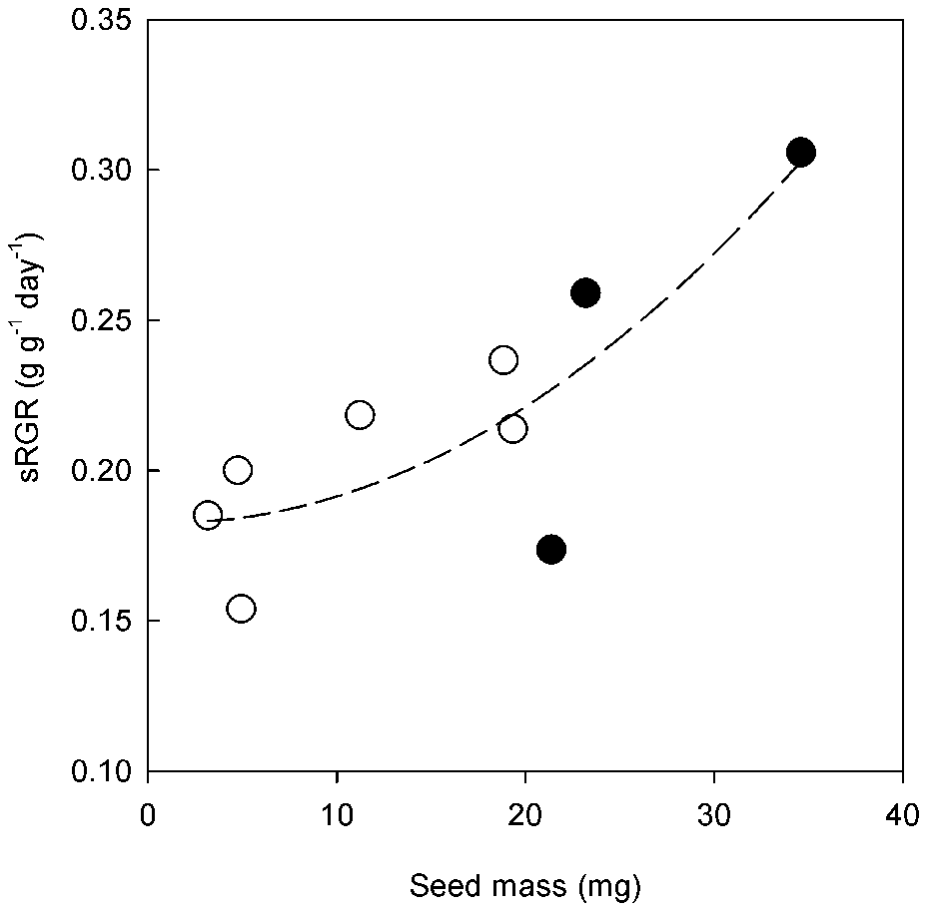
Relationship between size standardised RGR (sRGR) and seed mass. Regression slope for the relationship between sRGR and seed mass (F_2,6_ = 6.186, *P*<.05, R^2^ = 0.6734) for the three crop progenitors (closed symbols) and six wild species (open symbols).

RGR was calculated using classical and size-standardised methods, and these models showed contrasting results. Using the classical method there was no relationship between seed mass and RGR (Fig. S3), whereas a weak, positive relationship arose using the size-standardized method (F_2,6_ = 6.2, *P*<.05, Fig. 3). This positive relationship was largely driven by the high value of sRGR for *H. spontaneum*, and larger seed mass did not always translate to greater sRGR. In particular, *T. boeoticum* had a much lower sRGR than wild grain species (*A. crassa* and *A. tauschii*) with similar seed masses (Figs. 1 and 3). In comparison, the two other crop progenitors (*T. dicoccoides* and *H. spontaneum*), which had the largest seed masses, also had the highest sRGRs. The sRGR value of 0.31 for *H. spontaneum* was more than double that of the wild species with the lowest sRGR (*E. distans*) and 34% greater than the wild species with the highest sRGR (*A. crassa*). *T. dicoccoides* similarly showed growth advantages of 73% and 10% respectively over these two wild species.

### Components of RGR

**Figure 4:**
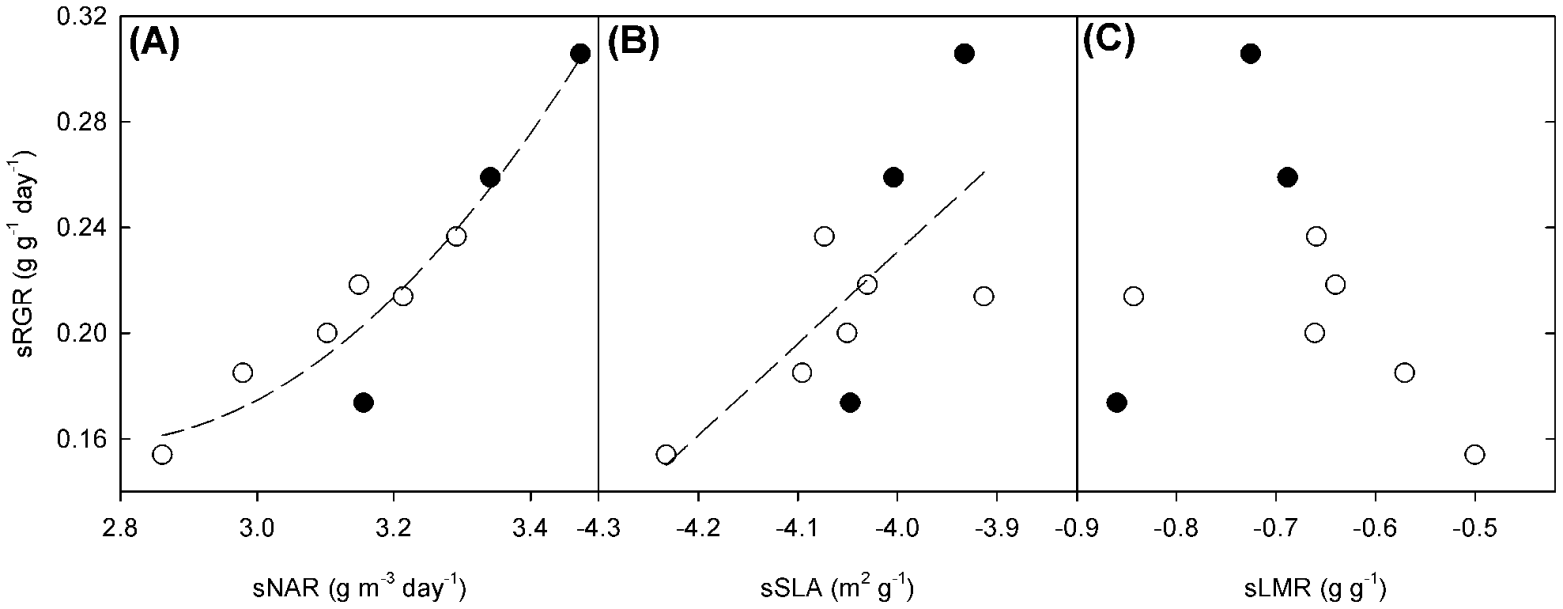
Relationship between sNAR, sSLA and sLMR and sRGR. Regression slopes for the relationships between (a) sNAR and sRGR (F_2,6_ = 31.98, *P*<.001, R^2^ = 0.914); (b) sSLA and sRGR (F_1,7_ = 6.781, *P*<.05, R^2^ = 0.492); and (c) sLMR and sRGR for the three crop progenitors (closed symbols) and six wild species (open symbols).

The best predictor of sRGR was sNAR (F_2,6_ = 20.1, *P*<.01, Fig. 4A), followed by sSLA (F_1,7_ = 6.8, *P*<.05, Fig 4B) whilst no relationship was found with sLMR (Fig. 4C). Supporting these findings, calculation of the contributions of sLMR, sSLA and sNAR to the variance in sRGR showed that sNAR made the largest contribution (82.5%), whilst sSLA (32.8%) made a small contribution, and sLMR a smaller, negative contribution (−15.3%). Normalization of the variance and covariance for each of these components of sRGR yields an “importance” value [30]. Importance values again showed sNAR to be the main driver of interspecific variation in sRGR (48.3%). However, sLMR was of equal importance to sSLA (26.9 *vs* 24.8% respectively). As expected from these results, sNAR correlates strongly with seed mass, whilst the relationship is weaker for the sLMR and sSLA components (see Fig. S4).

### Resilience to disturbance

**Figure 5:**
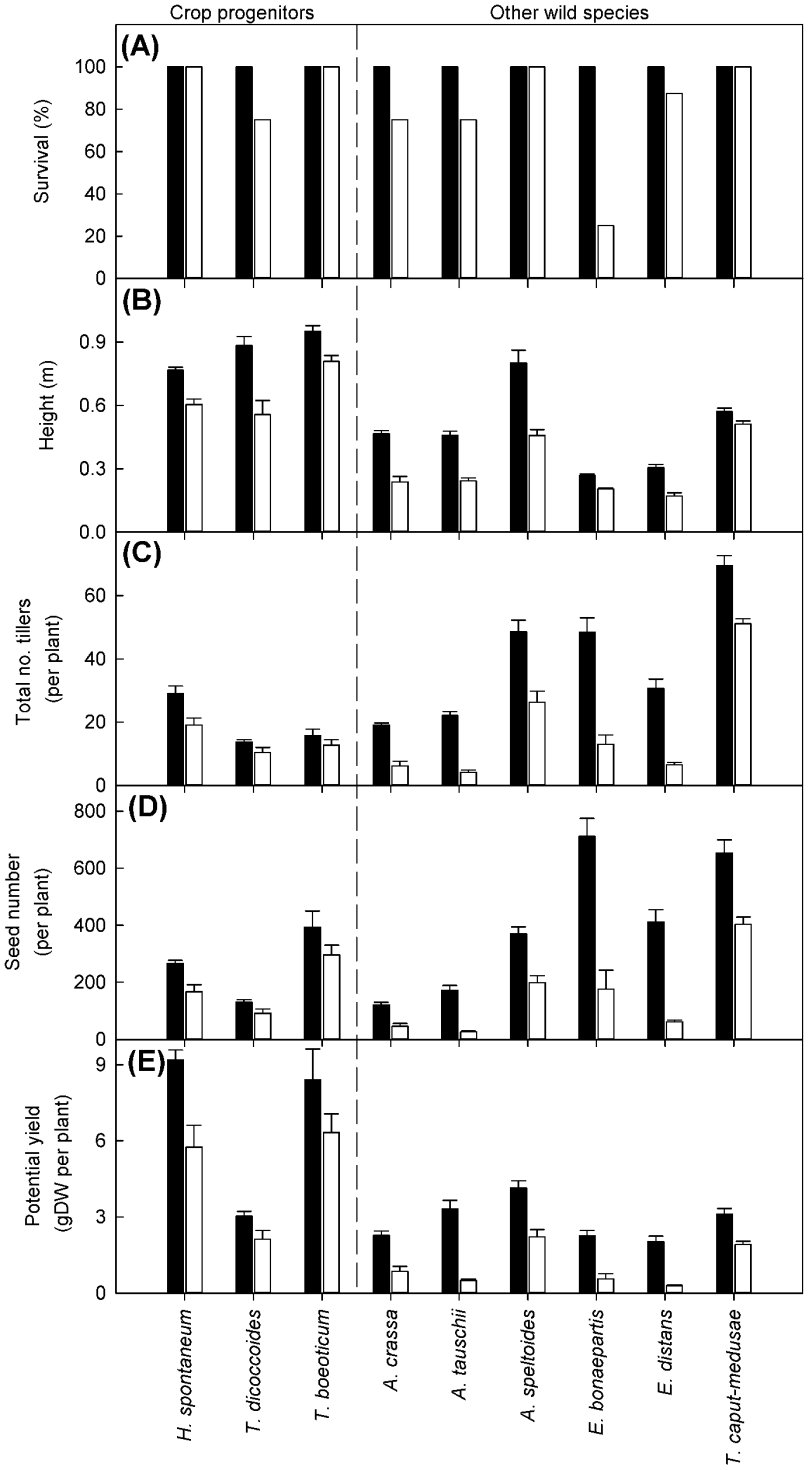
Impact of a defoliation treatment on plant survival, size and yield. Impact of defoliation treatment on (a) survival (%), (b) plant height, (c) number of tillers, (d) number of seeds, and (e) potential yield in crop progenitors and wild species. The defoliation treatment is shown by the white bar and the control treatment (no defoliation) is shown by the black. Data are means + SE of 8 replicates.

The defoliation treatment significantly reduced survival (F_1,134_ = 76.4, *P*<.001, Fig. 5A), with the magnitude of the response varying among species (F_8,135_ = 9.5, *P*<.001, Fig. 5A). The majority of the species defoliated showed 70–100% survival but, in *E. bonaepartis*, only 25% of the plants survived. Comparisons of survival between the crop progenitors and wild species showed no significant difference in the response to defoliation.

Defoliation significantly reduced the final height of plants (F_1,121_ = 103.4, *P*<.001, Fig. 5B), and the response varied between species from a 10% to 48% loss of height compared to the control treatment, leading to a significant interaction between species and treatment (F_8,113_ = 6.5, *P*<.001, Fig. 5B). Comparisons of plant height between the crop progenitors and wild species showed that, under the control treatment, the crop progenitors were significantly taller than the wild ones (t_1_ = 10.1, *P*<.001, Fig. 5B). However, there was no significant difference between the two types in the response of plant height to defoliation.

The defoliation treatment significantly decreased the number of tillers produced by each species (χ^2^_1_(N = 144)  = 287.2, *P*<.001, Fig. 5C). This varied between species from 19% in *T. boeoticum* to 81% in *A. tauschii*, leading to a significant species by treatment interaction (χ^2^_8_(N = 144)  = 134.3, *P*<.001, Fig. 5C). The wild species had a significantly greater number of tillers than the crop progenitors under the control treatment (z = 13.3, *P*<.001, Fig. 5C). The two types also showed a differential response to the defoliation treatment, with the decline in tiller number being greater in the wild species than crop progenitors (z = 7.1, *P*<.001, Fig. 5C). The full defoliation treatment led to, on average, a 62% decrease in the number of tillers in the wild species but only a 26% decline in the crop progenitors.

The period of time plants took to flower was extended significantly when a defoliation treatment was applied, with flowering taking 25–49% longer in the defoliated plants compared to the controls (χ^2^_1_(N = 144)  = 60.9, *P*<.001, data not shown). Once flowering was complete and seed set had begun, comparison of seed number between the control and defoliation treatment showed that the decrease in tiller number due to defoliation caused a large decline in seed number (χ^2^_1_(N = 144)  = 211.3, *P*<.001, Fig. 5D) and therefore potential yield (χ^2^_1_(N = 144)  = 119.4, *P*<.001, Fig. 5E). The proportion of tillers that set seed was not affected by defoliation (data not shown), so the decrease in seed number and potential yield was due to the reduction in tillers alone. The reduction in seed number due to defoliation varied between species (χ^2^_8_(N = 144)  = 118.4, *P*<.001, Fig. 5D) and was greater in the wild species compared to the crop progenitors (z = 6.6, *P*<.001, Fig 5D). The decrease in potential yield due to defoliation varied between species from 25% in *T. boeoticum* to 85% in *A. tauschii*, leading to a significant interaction between species and treatment (χ^2^_8_(N = 144)  = 16.1, *P*<.05, Fig. 5E). Comparison between the two types showed that, in the unperturbed control treatment, the crop progenitors were capable of producing a significantly larger yield than the wild species (z = 6.2, *P*<.001, Fig 5E). The two groups also showed a differential response to the defoliation treatment, with a significantly greater decline in yield in the wild species than crop progenitors (z = 2.8, *P*<.01, Fig 5E). The full defoliation treatment led to, on average, a 61% decrease in potential yield in the wild species, but only a 31% decline in the crop progenitors.

## Discussion

Results from the three experiments showed that crop progenitors have larger seed and seedling mass, earlier germination, and greater biomass and leaf area during early stages of vegetative growth than the other wild species known to have been gathered by pre-agricultural communities. Additionally, the greater seed mass was weakly but positively correlated with a higher sRGR in two of the three crop progenitors tested. The crop progenitors were more resilient in their response to defoliation and were taller at maturity, with a higher potential grain yield than the other wild species.

We hypothesize that this suite of functional traits observed in crop progenitors would have been advantageous in an anthropogenic environment. Initially, gathering would have occurred in the natural habitat of these wild plants and involved collection across a wide range of plant taxa. The gathering of wild plant seeds likely led to their dispersal in and around early human settlements. Seeds dispersed in this way would have been exposed to new selective pressures. Collected species that survived and flourished in such environments would have been those best able to survive disturbance and take advantage of greater levels of soil fertility. These species would also respond well to early attempts at cultivation. Enhanced competitive ability over smaller seedlings [36] and greater resilience to defoliation are of particular importance in this context [49], [50].

This suggestion is superficially similar to the so-called ‘dump heap hypothesis’ that crop plants originated from weeds associated with human refuse heaps and disturbed habitats surrounding pre-agricultural settlements (e.g. [51]–[53]; and subsequently discussed by, for example: Harlan [54], [55], Blumler and Byrne [56] and Abbo *et al*. [57]). It differs from it, however, in that we are not suggesting that crop progenitors were from naturally weedy habitats. Rather our hypothesis rests upon the more subtle differences between collected crop progenitors and other wild species that were also collected but never domesticated, and their relative competitive ability when introduced to a new environment. Our experiments focus specifically on the comparison between these collected species of Southwest Asia.

We argue that by out-competing other species less well adapted to these human-managed environments cereal crop progenitors became more abundant around human settlements and under early cultivation, and therefore more likely to be collected and harvested. This may have driven species selection in the early stages of plant domestication making human populations more dependent on a narrower range of species [18], [58], which then became the most likely candidates for, and successful products of, cultivation. Later, as wild progenitors were taken into cultivation, these same functional traits would have continued to offer competitive advantages, *within* cultivated species in deliberately sown, possibly weeded and/or manured cultivation plots.

### Larger size may confer a competitive advantage in crop progenitors

In this study, the crop progenitors had a larger seed mass than wild species that were not domesticated. Greater seed mass and seedling size enhance competitive ability [36] through a number of mechanisms. Under conditions of drought, burial or competition, survival is greater due to the longer initial hypocotyl or radicle, termed the ‘seedling size effect’ [37], [59], and we found a strong correlation between seed size and seedling size in experiment 1. If larger seedlings have lower RGR, as shown in previous studies [28], this is hypothesised to give a metabolic effect [37] where a species with a slower growth rate has a slower respiration rate and consumes metabolic resources more slowly. However, in this study and others [30], [31], RGR was size-dependent, and the relationship between size-corrected growth rate and seed mass was positive. Larger seeds have an additional benefit of having extra metabolic resources that may serve to better support carbon deficits, the ‘reserve effect’, where a greater amount of resources is left uncommitted at a given time after germination [37], [59]. This is of particular significance when defoliation occurs at a young age [49].

This study shows that larger seed size is weakly but positively associated with a higher RGR at a given plant size. A high sRGR offers larger seeds an advantage by allowing more rapid colonization of an environment. The crop progenitors *H. spontaneum and T. dicoccoides* had the largest seed sizes in this study and the corresponding highest sRGR, the relationship being stronger in *H. spontaneum*. However, the third crop progenitor, *T. boeoticum*, had a similar seed mass to the larger seeded *Aegilops* spp., but a much lower sRGR than expected from its seed size, which weakens the relationship. A useful future addition to this study would be to extend the work to the other primary domesticates including pulses such as pea, lentil, chickpea and bitter vetch to test whether the same relationships exist between seed size and functional traits related to competition and disturbance.

sNAR was the main driver of interspecific variation in sRGR, in contrast to other size-corrected studies [30], [42] where sSLA was most important. This dependence on sNAR may relate to high growth irradiance. In a meta-analysis by Shipley *et al*. [47], NAR was the most important driver of RGR in experiments conducted at a DQI of above 25 mol m^−2^ day^−1^; in our experiment the DQI was 43.6 mol m^−2^ day^−1^. Below 15 mol m^−2^ day^−1^ (low irradiance) SLA dominates, and between 15 and 25 mol m^−2^ day^−1^ SLA and NAR are of equal importance [47]. Furthermore, other studies have shown NAR to have increased importance at high irradiance [41], [60], [61]. In fact, there is a strong trade-off between NAR and SLA with changing light intensity [60]. The fast growing species in this study are likely to have a high photosynthetic capacity, which is realised under high irradiance. Photosynthetic rate and NAR are closely correlated, with photosynthesis in different species having different light saturation points, driving species differences in NAR at high light [60], [61].

Although large seeds may gain an advantage in seedling competition, small seeds have the advantage of being produced in much greater numbers for a given reproductive effort [27]. For example, under the control treatment in this study, the greatest number of seeds in the crop progenitors was 393 in *T. boeoticum,* whilst in the wild species the maximum was 711 in *E. bonaepartis*. Therefore, although large seeded species may be more competitive, they are often recruitment-limited [36]. However, as humans increasingly become the agents of dispersal for crop species, this limitation diminishes in importance, thereby removing an important cost from the evolutionary trade-off acting on seed size.

The advantages of large seed/seedling size mainly operate during the early stages of plant establishment and growth. However, we found that plant traits in established plants are also correlated with seed mass, albeit less strongly. The larger seed size and higher sRGR translate to mature plants with a taller stature and greater yield. Plant height, measured in the third experiment, differed between the two groups; crop progenitors were significantly taller overall, varying from 0.77 m to 0.95 m, whilst the wild species ranged from 0.27 to 0.80 m in height. As well as offering greater apparency in a landscape for human gatherers, this height advantage confers competitive ability through prior access to light, and may be particularly advantageous in conditions of high fertility where competitive plants are better equipped to take advantage of the higher nutrient availability [62], [63]. The ability of wild progenitors to exploit high fertility locations is expected to have been particularly advantageous in and around the human settlements, with nutrient enriched soils, to which they were transported by human gathering from the wild.

Fuller *et al.* [64] list three competing hypotheses to explain the observed increase in grain size during domestication (other than conscious human selection of larger grains within an existing population): (1) that larger grain size is a plastic response to the favourable soil conditions of cultivation, resulting in more fully developed grains [8]; (2) that larger grain varieties were adopted from elsewhere [8]; and (3) that larger size was an adaptive response to disturbance and/or deeper burial during cultivation [65]. Furthermore, genetics is likely to have played a significant role in the increase in seed size, with genetic control of grain size demonstrated not only in wheat [66], but also in other cereals e.g. rice [67], [68] and maize [69]. Our experiments provide empirical evidence for an association between grain size and other potentially adaptive plant characteristics and, in particular, indicate that increased grain size could reflect an adaptive response to both improved soil conditions and disturbance.

### Resilience to defoliation meant that yields were less impacted in crop progenitors

In this study, large seed mass was associated with substantial increases in potential yield; under the control treatment, the average yield per plant was 2.9 times greater in crop progenitors than wild species, which in itself may have been an important selection criterion for early farmers. Response to the defoliation treatment also differed significantly between the crop progenitors and wild species, with potential yield reduced more in the wild species. Although large seedlings are known to survive defoliation better, with higher subsequent growth rates [49], the plants in this study were beyond the seedling stage before the defoliation treatment was applied, so the response to defoliation cannot be attributed directly to seed mass.

Yield after defoliation was controlled by the number of tillers that re-sprouted; the number of tillers was significantly reduced by the defoliation treatment and this effect was much stronger in the wild species. Re-sprouting after the loss of practically all above-ground biomass requires surviving buds or other meristematic tissue, as well as reserves of carbohydrate and nutrients that can fund expansion of the first leaves of the new sprout [70], [71]. Re-sprouting is also related strongly to growth form, with grasses being strong re-sprouters [72]. This explains why the level of survival was high in our experiment. However, considerable between-species variation in the response to defoliation suggests that grasses may employ diverse strategies [73]. The smaller-seeded wild species used in this study could have a reduced ability to re-sprout after defoliation (resilience) because they recruit more resources into producing a larger soil seed bank [74], [75]. Alternatively, a smaller reduction in the tillering of crop progenitors could be an adaptation to grazing [76].

## Conclusions

Comparison of cereal crop progenitors with other wild grass species exploited by pre-agricultural societies in the Fertile Crescent of Southwest Asia revealed significant differences in functional traits related to competition and disturbance. Supporting our hypothesis (i), the crop progenitors had larger seeds and seedlings than the other wild species, and this was associated with faster germination. The larger seed size was also correlated with a greater size-corrected RGR for two of the three crop progenitors (ii). The height of the crop progenitors and the seed yield were greater at maturity, in agreement with hypothesis (iii) and, although the survival and ability to produce seeds did not differ between the crop progenitors and wild species [hypothesis (iv)], the crop progenitors were more resilient and seed yields were less impacted by defoliation. Hypotheses (i) and (ii) were linked to seed mass, whilst hypotheses (iii) and (iv) were more weakly associated. For the crop progenitors, this combination of traits related to competition and disturbance confers the ability to effectively exploit sites with high levels of fertility and disturbance, potentially allowing them to thrive around early pre-agricultural settlements while other collected species did not. The greater height and potential yield of these species would also have offered a highly apparent and more abundant food resource for gatherers. These same traits would have pre-adapted crop progenitors to the cultivated and managed field environment. Based on this evidence, we argue that the interaction of plant functional traits and ecological processes had the potential to exert a strong influence on the narrowing of the food resource base during the transition to agriculture in the Fertile Crescent. It would be interesting to make similar comparisons of the competitive ability of the progenitors of pulse crops and small-seeded grass crops in anthropogenic conditions, compared with other collected species in their areas of origin,

## Supporting Information

Figure S1 **Growth functions fitted to plots of ln-total plant mass against time.** Linear fits to ln-transformed dry weight per plant, in crop progenitors (a–c) and wild species (d–i). The crop progenitors were: (a) *H. spontaneum*, (b) *T. boeoticum*, (c) *T. dicoccoides*. The wild species were: (d) *A. crassa*, (e) *A. speltoides*, (f) *A. tauschii*, (g) *E. bonaepartis*, (h) *E. distans* and (f) *T. caput-medusae*. Data from experiment 2.
(DOCX)

Figure S2 **Relationship between dry weight, leaf area and seed mass.** Regression slopes for the relationship between (a) dry weight and seed mass (F = 19.001, d.f = 2,6, p = 0.0025, R^2^ = 0.8636); and (b) leaf area and seed mass (F = 14.32, d.f = 2,6, p = 0.005, R^2^ = 0.827) for the three crop progenitors (closed circle) and six wild species (open circle). Data from experiment 2.
(DOCX)

Figure S3 **Relationship between classical RGR and seed mass.** Relationship between classical RGR and seed mass for the three crop progenitors (closed circle) and six wild species (open circle). Data from experiment 2.
(DOCX)

Figure S4 **Relationship between sNAR, sSLA and sLMR and seed mass.** Regression slopes for the relationship between (a) sNAR and seed mass [(F = 12.988, d.f = 2,6, p = 0.007, R^2^ = 0.812)]; (b) sSLA and seed mass; and (c) sLMR and seed mass for the three crop progenitors (closed circle) and six wild species (open circle). Data from experiment 2.
(DOCX)

Table S1 **Details of species used in the experiments.** Table details whether species were a crop progenitor or wild species, the germplasm holding from where seed was obtained and the accession number in the collection. Germplasm holdings are: Leibniz Institute of Plant Genetics and Crop Plant Research (IPK) in Gatersleben, Germany; the National Small Grains Collection (NSGC) of the United States Department of Agriculture (USDA) at the University of Idaho R & E Center, Aberdeen, Idaho; and the Western Regional Plant Introduction Station (WRPIS) of the USDA, Pullman, Washington.
(DOCX)
