# Supplementary material for: Functional Traits Differ between Cereal Crop Progenitors and Other Wild Grasses Gathered in the Neolithic Fertile Crescent
Source: PLoS One. 2014 Jan 28;9(1):e87586. doi: 10.1371/journal.pone.0087586 (PMC3905035; doi:10.1371/journal.pone.0087586)
Supplement: Figure S1 — Growth functions fitted to plots of ln-total plant mass against time. Linear fits to ln-transformed dry weight per plant, in crop progenitors (a–c) and wild species (d–i). The crop progenitors were: (a) H. spontaneum, (b) T. boeoticum, (c) T. dicoccoides. The wild species were: (d) A. crassa, (e) A. speltoides, (f) A. tauschii, (g) E. bonaepartis, (h) E. distans and (f) T. caput-medusae. Data from experiment 2. (DOCX) [file pone.0087586.s001.docx]

**Figure S1.** **Growth functions fitted to plots of ln-total plant mass against time.**

Linear fits to ln-transformed dry weight per plant, in crop progenitors (a-c) and wild species (d-i). The crop progenitors were: (a) *H. spontaneum*, (b) *T. boeoticum*, (c) *T. dicoccoides*. The wild species were: (d) *A. crassa*, (e) *A. speltoides*, (f) *A. tauschii*, (g) *E. bonaepartis*, (h) *E. distans* and (f) *T. caput-medusae*. Data from experiment 2.
