# Supplementary material for: Functional Traits Differ between Cereal Crop Progenitors and Other Wild Grasses Gathered in the Neolithic Fertile Crescent
Source: PLoS One. 2014 Jan 28;9(1):e87586. doi: 10.1371/journal.pone.0087586 (PMC3905035; doi:10.1371/journal.pone.0087586)
Supplement: Figure S2 — Relationship between dry weight, leaf area and seed mass. Regression slopes for the relationship between (a) dry weight and seed mass (F = 19.001, d.f = 2,6, p = 0.0025, R2 = 0.8636); and (b) leaf area and seed mass (F = 14.32, d.f = 2,6, p = 0.005, R2 = 0.827) for the three crop progenitors (closed circle) and six wild species (open circle). Data from experiment 2. (DOCX) [file pone.0087586.s002.docx]

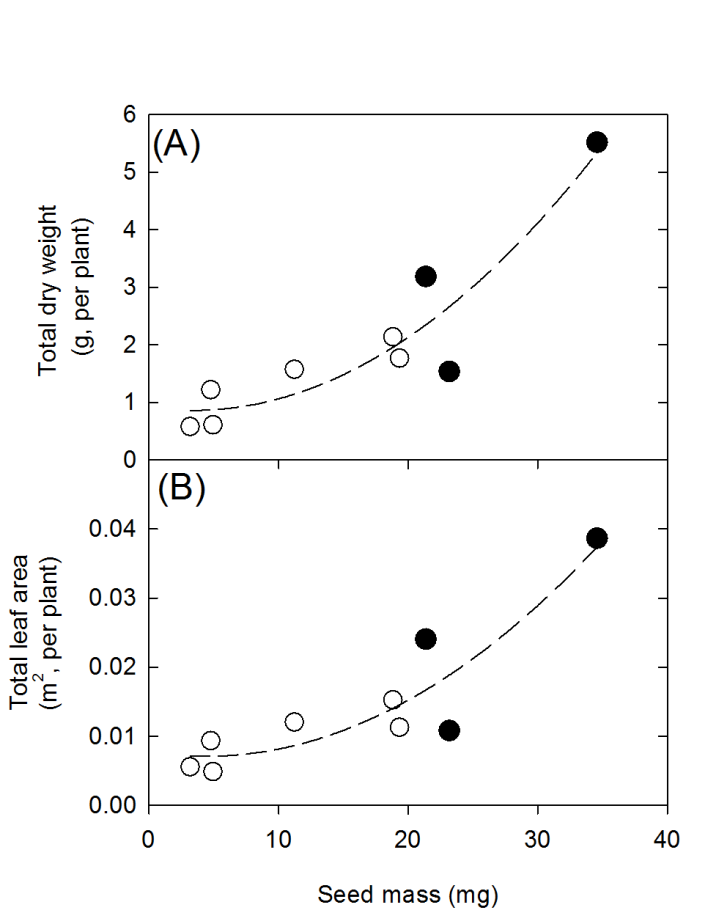


**Figure S2.** **Relationship between dry weight, leaf area and seed mass.**

Regression slopes for the relationship between (a) dry weight and seed mass (F=19.001, d.f=2,6, p=0.0025, R^2^=0.8636); and (b) leaf area and seed mass (F=14.32, d.f=2,6, p=0.005, R^2^=0.827) for the three crop progenitors (closed circle) and six wild species (open circle). Data from experiment 2.
