# Supplementary material for: Functional Traits Differ between Cereal Crop Progenitors and Other Wild Grasses Gathered in the Neolithic Fertile Crescent
Source: PLoS One. 2014 Jan 28;9(1):e87586. doi: 10.1371/journal.pone.0087586 (PMC3905035; doi:10.1371/journal.pone.0087586)
Supplement: Figure S3 — Relationship between classical RGR and seed mass. Relationship between classical RGR and seed mass for the three crop progenitors (closed circle) and six wild species (open circle). Data from experiment 2. (DOCX) [file pone.0087586.s003.docx]

**Figure S3**. **Relationship between classical RGR and seed mass.**

Relationship between classical RGR and seed mass for the three crop progenitors (closed circle) and six wild species (open circle). Data from experiment 2.
