# Supplementary material for: Functional Traits Differ between Cereal Crop Progenitors and Other Wild Grasses Gathered in the Neolithic Fertile Crescent
Source: PLoS One. 2014 Jan 28;9(1):e87586. doi: 10.1371/journal.pone.0087586 (PMC3905035; doi:10.1371/journal.pone.0087586)
Supplement: Figure S4 — Relationship between sNAR, sSLA and sLMR and seed mass. Regression slopes for the relationship between (a) sNAR and seed mass [(F = 12.988, d.f = 2,6, p = 0.007, R2 = 0.812)]; (b) sSLA and seed mass; and (c) sLMR and seed mass for the three crop progenitors (closed circle) and six wild species (open circle). Data from experiment 2. (DOCX) [file pone.0087586.s004.docx]

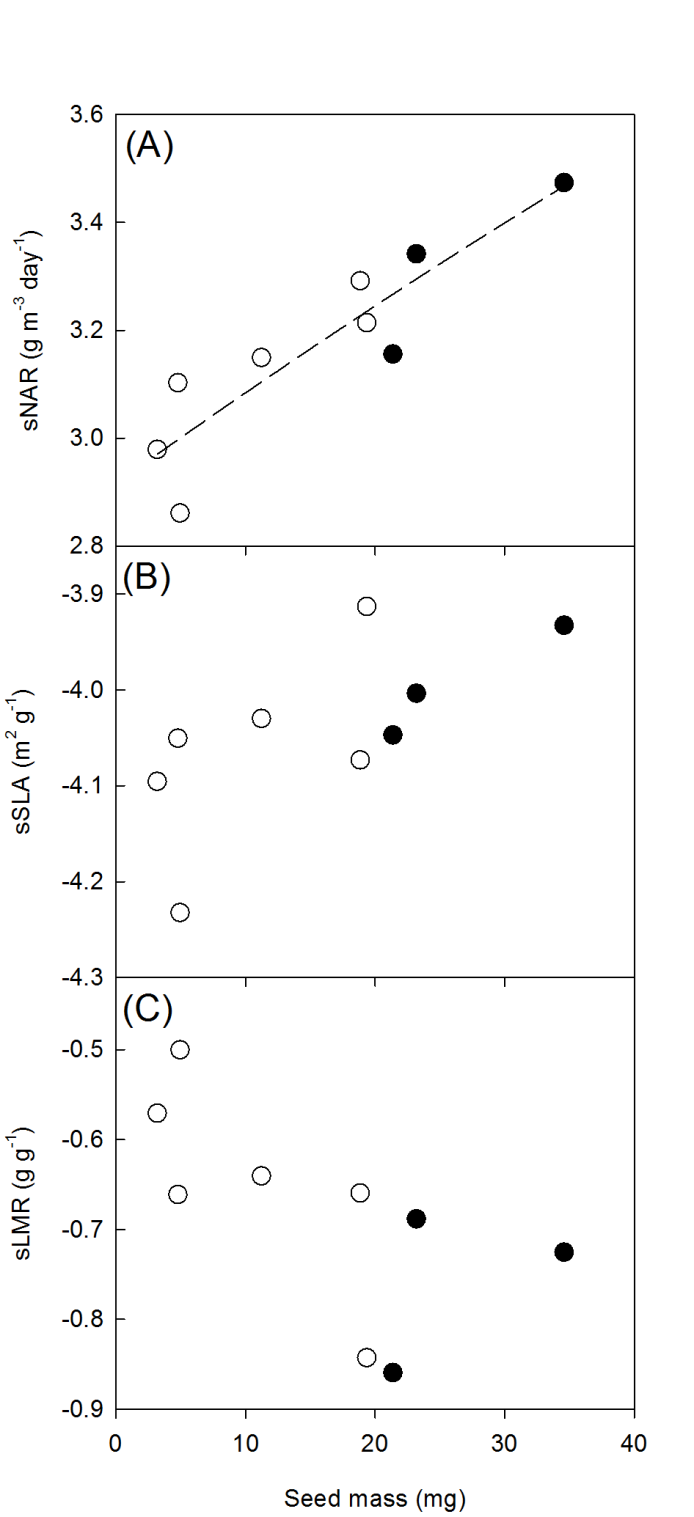


**Figure S4.** **Relationship between sNAR, sSLA and sLMR and seed mass.**

Regression slopes for the relationship between (a) sNAR and seed mass [ (F=12.988, d.f=2,6, p=0.007, R^2^=0.812)]; (b) sSLA and seed mass; and (c) sLMR and seed mass for the three crop progenitors (closed circle) and six wild species (open circle). Data from experiment 2.
