# Supplementary material for: Functional Traits Differ between Cereal Crop Progenitors and Other Wild Grasses Gathered in the Neolithic Fertile Crescent
Source: PLoS One. 2014 Jan 28;9(1):e87586. doi: 10.1371/journal.pone.0087586 (PMC3905035; doi:10.1371/journal.pone.0087586)
Supplement: Table S1 — Details of species used in the experiments. Table details whether species were a crop progenitor or wild species, the germplasm holding from where seed was obtained and the accession number in the collection. Germplasm holdings are: Leibniz Institute of Plant Genetics and Crop Plant Research (IPK) in Gatersleben, Germany; the National Small Grains Collection (NSGC) of the United States Department of Agriculture (USDA) at the University of Idaho R & E Center, Aberdeen, Idaho; and the Western Regional Plant Introduction Station (WRPIS) of the USDA, Pullman, Washington. (DOCX) [file pone.0087586.s005.docx]

**Table S1.** **Details of species used in the experiments.**

Table details whether species were a crop progenitor or wild species, the germplasm holding from where seed was obtained and the accession number in the collection. Germplasm holdings are: Leibniz Institute of Plant Genetics and Crop Plant Research (IPK) in Gatersleben, Germany; the National Small Grains Collection (NSGC) of the United States Department of Agriculture (USDA) at the University of Idaho R & E Center, Aberdeen, Idaho; and the Western Regional Plant Introduction Station (WRPIS) of the USDA, Pullman, Washington.

| Species | Crop progenitor / wild species | Source  (exp. 2) | Accession No. (exp. 2) | Source  (exp. 3) | Accession No. (exp. 3) |
| --- | --- | --- | --- | --- | --- |
| *Hordeum spontaneum*  K. koch. | Crop progenitor | IPK Gatersleben | HOR 9476/85 | IPK Gatersleben | HOR 13798 |
| *Triticum dicoccoides*  Koern. | Crop progenitor | NSGC (USDA) | PI 1428020 | IPK Gatersleben | TRI 18492 |
| *Triticum boeoticum*  Boiss. | Crop progenitor | IPK Gatersleben | TRI 17082 | IPK Gatersleben | TRI 17812 |
| *Aegilops crassa*  Boiss. | Wild species | NSGC (USDA) | PI 487286 | IPK Gatersleben | AE 299 |
| *Aegilops speltoides*  Tausch | Wild species | NSGC (USDA) | PI 174010 | IPK Gatersleben | AE 413 |
| *Aegilops tauschii*  Coss. | Wild species | NSGC (USDA) | PI 486266 | IPK Gatersleben | AE 248 |
| *Eremopyrum bonaepartis*  (Spreng.) Nevski | Wild species | WRPIS (USDA) | PI 227345 | IPK Gatersleben | GRA 789 |
| *Eremopyrum distans*  (K. Koch) Nevski | Wild species | WRPIS (USDA) | PI 193264 | WRPIS (USDA) | PI 193264 |
| *Taeniatherum caput-medusae*  (L.) Nevski | Wild species | WRPIS (USDA) | PI 577708 | IPK Gatersleben | GRA 866 |
